# Patient experiences and health system responsiveness in South Africa

**DOI:** 10.1186/1472-6963-9-117

**Published:** 2009-07-14

**Authors:** Karl Peltzer

**Affiliations:** 1 Health Promotion Research Unit, Social Aspect of HIV/AIDS and Health, Human Sciences Research Council, Pretoria, South Africa; 2 Department of Psychology, University of the Free State, Bloemfontein, South Africa

## Abstract

**Background:**

Patients' views are being given more and more importance in policy-making. Understanding populations' perceptions of quality of care is critical to developing measures to increase the utilization of primary health care services. Using the data from the South African *World Health Survey *(WHS), the current study aims to evaluate the degree of health care service responsiveness (both out-patient and in-patient) and comparing experiences of individuals who used public and private services in South Africa.

**Methods:**

A population-based survey of 2352 participants (1116 men and 1236 women) was conducted in South Africa in 2003, the WHS – as part of a World Health Organization (WHO) project focused on health system performance assessment in member countries.

**Results:**

Health care utilization was among those who attended in-patient care 72.2% attended a public and 24.3% a private facility, and of those who attended out-patient care 58.7% attended a public and 35.7% a private facility. Major components identified for out-patient care responsiveness in this survey were highly correlated with health care access, communication and autonomy, secondarily to dignity, confidentiality and quality of basic amenities, and thirdly to health problem solution. The degree of responsiveness with publicly provided care was in this study significantly lower than in private health care. Overall patient non-responsiveness for the public out-patient service was 16.8% and 3.2% for private care. Discrimination was also one of the principal reasons for non-responsiveness in all aspects of provided health care.

**Conclusion:**

Health care access, communication, autonomy, and discriminatory experiences were identified as priority areas for actions to improve responsiveness of health care services in South Africa.

## Background

The majority of South Africans depend on the public health sector for their health care needs [1]; the percentage of adults who reported that they have medical aid in the Demographic and Health Survey in 2003 was 14.2% [2]. Shisana et al. [3] found in a nationally representative survey that the majority (70%) indicated that they usually attended public health care services, while 23.3% attended private health care services, and a small proportion (0.1%) utilised traditional health practitioners. In many areas of South Africa, the Primary Health Care (PHC) facilities are the only available or easily accessible health service for local communities. As a result, PHC services, providers and facilities carry a large burden and responsibility for the provision of health care in South Africa. PHC is the basic mechanism for providing health care [4]. It was formally introduced in South Africa in April 1994 as the major principle for health care provision with the implementation of two policies, "Free health care for pregnant mothers and children under the age of six years" as well the "Universal Access to PHC for All South Africans" [4]. The Department of Health's strategic framework for 2002–2004 identifies improvements of quality of care as one of the four key challenges currently facing the health sector in South Africa [5]. Quality of care is concerned with the interface between provider and patients, between health services and community. A quality perspective changes the focus of health systems development from establishing structures to addressing what happens in the structures. Improving quality can, therefore, be regarded as a second phase of health care transformation in South Africa. The first phase was concerned with creating coherent health care structures and the second phase with ensuring quality of service delivery [5].

Variations in the perception of quality occur as a result of the heterogeneous nature of the definition of quality. Studies have pointed to variations in perception of quality by different socioeconomic groups as well as the environmental aspects such as the social, organisational and technological context of the service [6]. Van Vuuren and Botes [7] found among a culturally diverse population in an urban area in South Africa (greater Bloemfontein) that variables such as population group, age and employment status influence their attitudes towards professional health care. They further emphasise the importance of bringing these issues to the attention of health care policy makers. Peltzer [8] found in a community survey in rural South Africa a low acceptability of primary health care: 78% felt that the medical services are poor. Bediako, Nel and Hiemstra [9] found among hospital and out-patients in the North-West Province that more than half of the patients (56.8%) were not satisfied with the availability of medicines and other supplies. Approximately two thirds of patients (65.2%) did not know about the quality of telephone services rendered. There was a high level of dissatisfaction (63.1%) among patients regarding accessing doctors after hours. Most patients were satisfied with the general attitude of health workers (62.1%) but 21.2% were dissatisfied. De Jager and Du Plooy [10] found among in- and out-patients in a provincial hospital in Gauteng significant differences between in- and out-patients. Personal safety and cleanliness of facilities were regarded as the most important variables in the assurance and tangibility dimensions. The level of satisfaction was the highest for clear information and communication at an understandable level in the tangibility and assurance categories, respectively. The South African Department of Health [2] found that there was an increase in the percentage of adults who expressed dissatisfaction with all types of health services, except for traditional healers, comparing the Demographic and Health Survey (DHS) of 1998 and 2003. Generally, the results show that considerably more people are dissatisfied with the services rendered in hospitals, both public (23.3%) and private (11.6%). Even the levels of dissatisfaction with the services rendered by solo practitioners in the private sector (7.9%) seem to be on the increase during the period between the surveys. The major reasons for dissatisfaction with the public sector hospitals and community health centres were long waiting times (41.5% and 38.1% respectively), staff attitudes (22.8% and 25.9% respectively), non-availability of prescribed medication (15.8% and 17.7% respectively) and shortages of staff (doctors/pharmacists). Major reasons for dissatisfaction in the private hospital/clinic sector and private doctor were also long waiting times (26.7% and 7.4% respectively), staff attitude (18.0% and 7.1% respectively), and cost (15.2% and 24.8% respectively) [2]. Myburgh, Solanki, Smith and Lalloo [11] used a 1998 national population-based survey and found that both race and socio-economic status (SES) were significant predictors of levels of satisfaction with the services of the health care provider; White and high SES respondents were about 1.5 times more likely to report excellent service compared with African Black and low SES respondents, respectively.

Patients' views are being given more and more importance in policy-making. Understanding populations' perceptions of quality of care is critical to developing measures to increase the utilization of primary health care services.

A population-based survey was conducted in South Africa in 2003, the WHS – as part of a WHO project focused on health system performance assessment in member countries. Among the surveyed aspects was the evaluation of health care provision, based on the concept of "responsiveness" [12]. Using the data from the South African WHS, the current study aims to evaluate the degree of responsiveness with provided health care (both out-patient and in-patient), and comparing the experiences of individuals who used public and private services in South Africa.

## Methods

### Sample and procedure

The country sample (n = 2352) was nationally representative and probabilistically selected using a multistage cluster design. All respondents were selected using a Kish table for selection within a household. The study included: (1) all individuals who had been hospitalized in the previous five years (stayed overnight in a hospital or other type of long-term care facility), and (2) among those who had not been hospitalized in the previous five years, all individuals who had used an out-patient health service in the past 12 months. Only the participants who had used an in-patient and/or out-patient health services were requested to complete the responsiveness questions. The number of responses obtained was a function of the overall response rate as well as the rate of service utilization in the previous 12 months for out-patient and 5 years for in-patient services. More detailed information about the World Health Survey is available on its website . To adjust for the population distribution as represented by the UN Statistical Division and for non-response, post-stratification corrections were made to the sampling weights.

Participants were interviewed face-to-face by lay people with at least a high school-level education; interviewers were trained in a week-long course using a standard manual and audiovisual aids as well as role-plays. Practice field interviews were reviewed by supervisors before actual data collection. All questionnaires were translated into major languages in South Africa and back-translated using a standard WHO protocol. The quality of translations was independently verified by bilingual experts before field implementation. Informed consent was obtained from all respondents and the study was cleared by ethics review committees.

### Outcome measures

The questionnaire included in this analysis used the health system responsiveness module. Responsiveness relates to patient's experiences with the health system, with a focus on the interpersonal aspects of the care, and differs from patient satisfaction, a construct that reflects people's expectations in addition to their experiences [13].

Questions covered the following aspects: traveling time to the health care provider (item wording: For your last visit [or hospital stay], how would you rate the traveling time to the health care provider [or hospital]?); waiting time before being attended to; being greeted and talked to respectfully; respect for intimacy during physical examination and care; clarity of explanations by the health care providers; availability of time to ask questions about the health problem or treatment; possibility of obtaining information on other types of treatment or tests; participation in decision-making on the health care or treatment; patient's freedom to speak privately with the health professionals; personal information kept confidential; freedom to choose the health care provider; inside the facility cleanliness including toilets; and available space in waiting and examination rooms. For participants who received in-patient care, two additional aspects were included: ease in receiving visits by family members (item wording: For your last hospital stay, how would you rate the ease of having family and friends visit you? and contact with the outside world) [13]. Response options for these responsiveness items were 1 = very good, 2 = good, 3 = moderate, 4 = bad, and 5 = very bad. These item responses were dichotomised into "1" and "2" = 1, and 3–5 = 0. In addition, there were three items for both out-patients and in-patients related to the health professional's skills (item wording for out-patients: In your opinion was the health care provider's skill adequate for your treatment? and item wording for in-patients: In your opinion, was the skill of the health care providers adequate for your treatment?), availability of medicines, and adequacy of equipment in the care; response options for these items were "yes" or "no". Cronbach alpha for the 16 items out-patients responsiveness scale was .89 for this sample and for the 18 items in-patient responsiveness scale .90 for this sample. Further, participants were asked whether they felt they had been treated worse by the health care providers (whether they felt discriminated) for on any of the following reasons: sex, age, lack of money, social class, ethnic group or skin color, type of illness, or nationality. Response options for these 7 items were "yes" or "no" [13].

### Data analysis

The first stage of this work included a descriptive analysis of the degree of responsiveness based on a set of variables that expressed the user's degree of experience, according to five response levels (1 = very good to 5 = very bad). For each of the items, the degree of responsiveness was estimated by the percentage of "good" or "very good" answers and the percentage of positive answers to three other dichotomous variables (yes or no), related to the health professional's skills, availability of medicines, and adequacy of equipment in the care. Percentages of patients' experiences were analyzed by "type of care" (out-patient or in-patient) and by public and private health care service utilization [13].

To explain total variation in the data set using a smaller number of factors, the second stage of the analysis used principal component analysis with varimax rotation.

Logistic regression was conducted for public versus private health care provider, and linear regression for the total responsiveness scale (16 responsiveness items for out-patients and 18 responsiveness items for in-patients), separately for public and private health care utilization. Demographic variables included sex, two age groups (18 to 39 and 40 years and above), two educational groups (up to primary school completed and above) [13], discriminatory experiences were summed up and converted into a binary variable with 1 = indicating any of the six discriminatory experiences and 0 = no such experiences.

For the statistical analysis the data were weighted according to the sampling design using Stata software version 10.0 (Stata Corporation, College Station, Texas, USA).

## Results

The final sample included 2352 participants (1116 men and 1236 women) with a mean age of 37.6 years (SD = 14.3), range 18 to 97 years; the household response rate was 80% and the individual response rate was 90%. Average item missing rates were less than 1% (range 0–1.8%) for the responsiveness questions. Of the 2352 interviewees, 656 (28.3%) reported in-patient care in the five years prior to the survey. Of these, 72.2% attended a government public facility, 24.3% a privately operated health facility, 1.3% an NGO and 2.4% other. Among the participants, 449 (19.4%) had received outpatient care at least once in the year prior to the interview. Of these, 58.7% attended a government public facility, 35.7% a privately operated health facility, 0.9% an NGO and 4.7% other. Overall, 5.1% reported to have health insurance. The type of the last out-patient health care provider visited was 52.6% medical doctor, 36.9% nurse, 5.5% dentist, 2.5% physiotherapist and 2.4% other, none indicated traditional health practitioner.

According to the evaluation of out-patient care (see Table 1), "*waiting time for care*" showed the lowest degree of responsiveness (51.2%) among all the areas analyzed. While, the aspects related to health professionals' skills (92.3%), adequate equipment (91.6%), adequate availability of medicines received (87.1%), and respect for intimacy during physical examination (77.9%) had the highest responsiveness scores. The percentage of users who gave "good" or "very good" ratings was lower among users of the public health care system for all aspects studied, both for out-patient and inpatient care. The percentage of users who rated their experiences with "bad" or "very bad" was 16.8% for public and 3.2% for private out-patient care (see Table 1).

**Table 1:** Last visit responsiveness in percent by public or private out-patient care (n = 424, excluding NGO and other: n = 25)

**Out-patient care**	Total (n = 424)	Public (n = 267)	Private (n = 157)	OR (95% CI)
Age (Mean)	37.6	41.0	36.6	Public = 1.00 Private = 1.02 (1.01–1.04)**

Female patient	61.6	69.1	49.2	Public = 1.00 Private = 2.31 (1.49–3.59)***

Education (Mean, range 1–7)	3.8	3.3	4.7	Public = 1.00 Private = 0.56 (0.48–0.66)***

Seen by medical doctor	53.5	36.6	81.7	Public = 1.00 Private = 0.13 (0.08–0.22)***

Time to get to facility in minutes (Mean)	29.3	33.5	21.0	Public = 1.00 Private = 1.02 (1.00–1.04)*

Provider fees (Mean in Rand)	37.5	16.1	150.1	Public = 1.00 Private = 0.98 (0.97–0.99)**

Medicines costs (Mean in Rand)	16.2	6.5	38.1	Public = 1.00 Private = 0.97 (0.94–1.00)*

**Patient satisfaction**				

1. Traveling time to the health care provider	64.6	54.3	81.0	Public = 1.00 Private = 0.28 (0.17–0.46)***

2. Waiting time	51.2	36.2	75.4	Public = 1.00 Private = 0.19 (0.12–0.30)***

3. Being greeted and talked to respectfully	69.2	59.7	84.7	Public = 1.00 Private = 0.81 (0.26–2.48)

4. Respect for intimacy during physical examination	73.6	65.2	87.2	Public = 1.00 Private = 0.60 (0.18–1.99)

5. Clarity of explanations	73.2	63.6	88.9	Public = 1.00 Private = 0.22 (0.12–0.39)***

6. Time for questions	64.5	52.7	83.7	Public = 1.00 Private = 0.22 (0.13–0.37)***

7. Possibility of obtaining information on other types of treatment	61.0	49.2	80.2	Public = 1.00 Private = 0.24 (0.15–0.39)***

8. Participation in treatment decision-making	58.0	48.5	73.5	Public = 1.00 Private = 0.34 (0.21–0.54)***

9. Privacy with health professionals	68.7	59.5	83.6	Public = 1.00 Private = 0.29 (0.17–0.49)***

10. Confidentiality of personal information	78.4	71.1	89.7	Public = 1.00 Private = 0.28 (0.15–0.54)***

11. Freedom to choose health care provider	56.9	42.2	80.8	Public = 1.00 Private = 0.17 (0.11–0.28)***

12. Cleanliness inside the health facility	72.7	62.6	89.1	Public = 1.00 Private = 0.21 (0.11–0.38)***

13. Available space in waiting and examination rooms	63.3	51.1	83.1	Public = 1.00 Private = 0.21 (0.13–0.36)***

14. Satisfactory health care provider skills	92.3	89.1	97.7	Public = 1.00 Private = 0.32 (0.13–0.81)*

15. Adequate equipment	91.6	89.5	94.9	Public = 1.00 Private = 0.52 (0.24–1.13)

16. Adequate availability of medicines	87.1	84.6	91.0	Public = 1.00 Private = 0.52 (0.31–1.08)

Total mean responsiveness (range 0–16)	11.4	9.8	13.6	Public = 1.00 Private = 0.78 (0.73–0.83)***

***P < .001; **P < .01; *P < .05

Among all the aspects of in-patient care responsiveness (Table 2), the lowest percentage of responsiveness was attributed to "*freedom to choose the health care provider*" (55.7%), while areas related to adequate health care provider skills, equipment, and availability of medicines had the highest health system responsiveness.

**Table 2:** Last visit responsiveness in percent by public and private in-patient care (n = 633, excluding NGO and other: n = 23)

**In-patient care**	Total (n = 633)	Public (n = 472)	Private (n = 161)	OR (95% CI)
Age (Mean)	38.9	38.7	39.4	Public = 1.00 Private = 0.99 (0.98–1.05)

Female patient	60.4	63.3	51.7	Public = 1.00 Private = 1.61 (1.49–3.59)*

Education (Mean, range 1–7)	3.9	3.5	5.1	Public = 1.00 Private = 0.40 (0.34–0.48)***

Time to get to facility in minutes	37	42	26	Public = 1.00 Private = 1.02 (1.00–1.03)*

Provider fees (in Rand)	246	44	907	Public = 1.00 Private = 0.99 (0.99–1.00)***

Medicines costs (in Rand)	78	9	299	Public = 1.00 Private = 0.99 (0.98–0.99)***

Tests	32	7	115	Public = 1.00 Private = 0.99 (0.99–1.00)**

Transport	37	41	24	Public = 1.00 Private = 1.00 (0.99–1.01)

Number of people slept in same room (Mean)	5.8	6.6	3.5	Public = 1.00 Private = 1.22 (1.06–1.40)**

**Patient satisfaction**				

1. Traveling time to the health care provider	67.8	61.2	87.5	Public = 1.00 Private = 0.23 (0.14–0.38)***

2. Waiting time	63.3	55.2	87.1	Public = 1.00 Private = 0.18 (0.11–0.31)***

3. Being greeted and talked to respectfully	70.6	63.3	92.0	Public = 1.00 Private = 0.15 (0.08–0.28)***

4. Respect for intimacy during physical examination	77.9	72.1	94.5	Public = 1.00 Private = 0.14 (0.07–0.30)***

5. Clarity of explanations	71.0	63.9	91.7	Public = 1.00 Private = 0.16 (0.08–0.31)***

6. Time for questions	63.0	55.0	86.4	Public = 1.00 Private = 0.19 (0.11–0.33)***

7. Possibility of obtaining information on other types of treatment	61.2	53.4	84.6	Public = 1.00 Private = 0.21 (0.13–0.35)***

8. Participation in treatment decision-making	60.6	50.4	90.6	Public = 1.00 Private = 0.11 (0.06–0.19)***

9. Privacy with health professionals	69.5	62.3	91.0	Public = 1.00 Private = 0.16 (0.09–0.30)***

10. Confidentiality of personal information	76.9	71.3	93.0	Public = 1.00 Private = 0.19 (0.10–0.36)***

11. Freedom to choose health care provider	55.7	45.8	84.4	Public = 1.00 Private = 0.11 (0.09–0.26)***

12. Cleanliness inside the health facility	72.6	65.8	92.6	Public = 1.00 Private = 0.15 (0.08–0.30)***

13. Available space in waiting and examination rooms	67.8	60.0	90.9	Public = 1.00 Private = 0.12 (0.07–0.22)***

14. Satisfactory health care provider skills	93.7	92.3	97.8	Public = 1.00 Private = 0.29 (0.09–0.88)***

15. Adequate equipment	91.8	89.9	97.2	Public = 1.00 Private = 0.26 (0.10–0.72)**

16. Adequate availability of medicines	89.7	87.8	95.3	Public = 1.00 Private = 0.44 (0.22–0.86)*

17. Ease of receiving visitors	73.9	67.1	94.1	Public = 1.00 Private = 0.13 (0.06–0.26)***

18. Ease of staying in contact with outside world	62.4	52.9	90.3	Public = 1.00 Private = 0.12 (0.08–0.88)***

Total mean responsiveness (range 0–18)	12.9	11.7	16.3	Public = 1.00 Private = 0.70 (0.64–0.77)***

***P < .001; **P < .01; *P < .05

A significant proportion of out-patient care users experienced discrimination for the following reasons: 11.9% reported feeling they had been treated worse than others because of lack of money and 9.3% because of their social class. Among users of the public services these figures were 15.9% and 13.3%, respectively. Of all users, 6.4% reported they had been treated worse because of their skin colour. Interviewees who had been hospitalized in the previous five years reported lower discrimination rates than out-patients, with "lack of money", race and "social class" as major factors. Users of in-patient and out-patient public health care reported significantly higher discrimination rates than private health care patients (see Table 3 and 4).

**Table 3:** Percentage of patients who experienced some type of discrimination by public and private out-patient care

Reason for discrimination	Total	Public	Private	OR (95% CI)
**Out-patient care**				

Sex	3.3	4.9	0.7	

Age	5.9	8.8	1.3	

Lack of money	11.9	15.9	5.3	

Social class	9.3	13.3	3.0	

Race	6.4	7.8	4.2	

Type of illness	6.3	9.0	2.0	

Nationality	4.3	5.7	2.1	

Total (Mean, range 0–7)	0.44	0.57	0.21	Private = 1.00 Public = 1.49 (1.09–2.03)*

**Table 4:** Percentage of patients who experienced some type of discrimination by public and private in-patient care

Reason for discrimination	Total	Public	Private	OR (95% CI)
Sex	2.2	2.8	0.3	

Age	3.1	3.9	0.3	

Lack of money	8.2	10.4	1.5	

Social class	6.8	8.6	1.7	

Race	7.0	8.3	3.2	

Type of illness	4.4	5.6	0.6	

Nationality	2.1	2.3	1.6	

Total (Mean, range 0–7)	0.35	0.43	0.11	Private = 1.00 Public = 1.80 (1.17–2.76)**

Principal component analysis found for out-patient care responsiveness three main factors explaining 58.5% of the variance and for in-patient care responsiveness four factors explaining 62.9% of the variance. The three factors for out-patient care responsiveness included 1) six items with two each on time, communication and autonomy (explaining 38.6% of the variance), 2) six items with each two items on dignity, confidentiality and quality of basic amenities (explaining 12.5% of the variance), and 3) three items on health problem solution (explaining 7.4% of the variance). The four factors for the in-patient care satisfaction included 1) six items with two each on communication, autonomy and confidentiality (explaining 37.1% of the variance), 2) four items with two each on time and dignity (explaining 11.7% of the variance), 3) three items on health problem solution (explaining 8.0% of the variance), and 4) four items with two each on quality of basic amenities and access to family and community support (explaining 6.2% of the variance). The overall responsiveness score was for out-patients 67% and for in-patients 68% (see Table 5).

**Table 5:** Principal component analysis with varimax rotation of health care responsiveness by out-patient and in-patient care (only items loading .40 or more are recorded)

		Out-patient experiences			In-patient experiences			
	Frequency (%)	Factor 1	Factor 2	Factor 3	Factor 1	Factor 2	Factor 3	Factor 4

	Out-patient = OP In-patient = IP	Time/Communication/Autonomy	Dignity/Confidentiality/Basic amenities	Health problem solution	Communication/Autonomy/Confidentiality	Time/dignity	Health problem solution	Basic amenities/ support

Traveling time to the health care provider	Time OP = 57.9	.40				.69		
		
Waiting time	IP = 65.6	.49				.76		

Being greeted and talked to respectfully	Dignity		.55			.60		
		
Respect for intimacy during physical examination	OP = 71.4 IP = 74.3		.72			.49		

Clarity of explanations	Communication	.73			.73			
		
Time for questions	OP = 68.9 IP = 67.0	.80			.77			

Possibility of obtaining information on other types of treatment	Autonomy OP = 59.5 IP = 60.9	.79			.76			
		
Participation in treatment decision-making		.80			.70			

Privacy with health professionals	Confidentiality		.53		.70			
		
Confidentiality of personal information	OP = 73.6 IP = 73.2		.58		.53			

Cleanliness inside the health facility	Quality of basic amenities		.80	.55				.66
		
Available space in waiting and examination rooms	OP = 68.0 IP = 70.2		.72					.68

Satisfactory health care provider skills	Health problem solution OP = 90.3 IP = 91.7			.78			.85	
		
Adequate equipment				.88			.89	
		
Adequate availability of medicines				.74			.76	

Ease of receiving visitors	Support IP = 68.2							.67
		
Ease of staying in contact with outside world								.49

Summary	OP = 67 IP = 68							

Variance (%)		38.6	12.5	7.4	37.1	11.7	8.0	6.2

The results of the multivariate analysis of the joint influence of sex, age group, formal education, public versus private health care, and discrimination experience on health care responsiveness are presented in Table 6. Only private health service and lower discrimination experienced were consistently associated with the total patient satisfaction score.

**Table 6:** Multivariate linear regression of demographic and health variables on total patient satisfaction [Dependent Variable: Ambulatory or in-patient responsiveness]

	Out-patients		In-patients	
	Total patient satisfaction		Total patient satisfaction	

	Coef. (CI 95%)	P	Coef. (CI 95%)	P

Sex				
Male	-.21 (-1.02–0.61)	0.61	-.43 (-1.17-.30)	0.24
Female				

Age				
18–39	.04 (-.83 – 0.91)	0.93	.14 (-.59-.86)	0.71
40 and more				

Education				
1–3	.23 (-.69–1.14)	0.62	.77 (-.08–1.62)	0.07
4–7				

Form of payment				
Public	-3.20 (-4.07–-2.33)	.000	-3.95 (-4.73–-3.18)	0.000
Private				

Discrimination				
Yes	-3.90 (-5.03–-2.78)	.000	-3.99 (-5.02–-2.95)	0.000
No				

## Discussion

The study conducted among a nationally representative sample in South Africa found that of those who attended in-patient care 72.2% attended a public and 24.3% a private facility, and of those who attended out-patient care 58.7% attended a public and 35.7% a private facility, none indicated traditional health practitioner. Similarly, Shisana et al. [3] found in a nationally representative study that the majority (70%) indicated that they usually attended public health care services, 23.3% attended private health care services, and 0.1% utilised traditional health practitioners.

The international comparison of health care responsiveness using the same measures and analysis found that overall South Africa (67% for out-patients and 68% for in-patients) had much lower responsiveness than Brazil (80% for out-patients and 76% for in-patients) and Israel and 14 European countries (81% and higher) for both out-patient and in-patient care. Looking at different components of responsiveness, South Africa scored particularly low on waiting time (58% for out-patients and 66% for in-patients) and autonomy (60% for out-patients and 61% for in-patients) care compared to Brazil (65% and 69% for out-patient and in-patient experiences respectively for waiting time and 70% and 66% respectively for autonomy), Israel (69% and 77% for waiting time and 80% and 79% for autonomy) and European countries (72% and 81% for waiting time and 83% and 72% for autonomy. The relative rankings of the domains among out-patients were similar in South Africa and Brazil, with the three highest rankings on confidentiality, dignity and communication, and the three lowest on waiting time, autonomy and quality of basic amenities. Regarding relative rankings on the domains among out-patients rankings were similar in South Africa and Brazil, with quality of basic amenities ranking third, after dignity and confidence. Rankings for European countries were similar, with the exception that quality of basic amenities was ranked higher than in middle income countries (Brazil and South Africa) (see Table 7) [13-15].

**Table 7:** Health care responsiveness (percentage of respondents who responded either "good" or "very good") comparisons across countries [13-15]

	Out-patient experiences				In-patient experiences			
	South Africa	Brazil	Israel	European countries*	South Africa	Brazil	Israel	European countries*

Time	58	65	69	72	66	69	77	81

Dignity	71	93	92	90	74	90	90	89

Communication	69	81	87	87	67	76	87	82

Autonomy	60	70	80	83	61	66	79	72

Confidentiality	74	90	88	89	73	80	83	82

Quality of basic amenities	68	80	90	91	70	80	60	87

Support					68	70	91	92

Summary	67	80	83	87	68	76	81	83

*European countries included were: Austria, Belgium, Denmark, Finland, France, Germany, Greece, Ireland, Italy, Luxemburg, Netherlands, Portugal, Sweden, and United Kingdom

Principal component analysis found in this study that responsiveness in out-patients included as major factors waiting time/communication/autonomy followed by dignity/confidentiality/basic amenities, and for in-patients communication/autonomy/confidentiality, waiting time/dignity and lastly quality of basic amenities/support. Similarly, Valentine, Darby and Bonsel [16] found from general population surveys of "health systems responsiveness" in 41 countries that most respondents selected prompt attention as the most important domain. Dignity was selected second, followed by communication. Access to social support networks was identified as the least important domain. The factor solutions from this study did not confirm the domain structure of 7 domains of previous studies [17]. This study underlines different clustering patterns of responsiveness for out-patients and in-patients in South Africa, in the Brazilian WHYS study [13] and in a study in Taiwan that found five factors (respect, access, confidentiality, basic amenities, and social support) [18]. For example, "autonomy" was in this and the Taiwanese study [18] not conceptualized as a unique domain. Further, studies are needed to identify the structure of health systems responsiveness domains in developing countries.

Regarding health care provider skills among out-patients in this study similar results were found between this study and the Brazilian WHS: 92.3% and 92.9% respectively, adequate equipment 91.3% and 91.6% respectively, and adequate availability of medicines 80.8% and 87.1%, and also among inpatients 91.3% and 93.7% for satisfactory health care provider skills, 92.3% and 91.8% for adequate equipment, and 92.9 and 89.7 for availability of medicine [13].

Major components identified for out-patient care responsiveness in this survey were highly correlated with health care access, communication and autonomy, secondarily to dignity, confidentiality and quality of basic amenities, and thirdly to health problem solution. Thus, from the perspective of health service users in South Africa, health care responsiveness was primarily related to health care access, communication and autonomy. Each of the three components got the lowest responsiveness ratings (59%–77%) compared to the other components (dignity, confidentiality, basic amenities and health problem solution) (76%–84%).

The degree of responsiveness with publicly provided care was in this study significantly lower than in private health care; a finding which was also found in local studies [2,8] and in the Brazilian WHS [13]. Overall lack of responsiveness for the public out-patient service was 16.8% in this study, which is lower to the DHS survey (23.3%) measuring patient dissatisfaction [2]. Both studies were conducted in the same year, 2003. Possible explanations for the above differences may lie in the better quality of private services or that expectations are already high among the population, both for users and nonusers, and because the different measures used in terms of getting a lower score with the responsiveness measure as compared to a dissatisfaction measure. In this study 15.4% of public out-patients were dissatisfied with the availability of medicines, which seem lower than in some local studies, 56.8% [9]. In multivariate regression analysis sex, age and educational level were not found to be associated with health care responsiveness unlike in some other studies [7,11,13,17].

Another problem identified in this study and also confirmed in the Brazilian WHS [13] was the high percentage of individuals who felt discrimination, regardless of public or private health care. Discrimination was also one of the principal reasons for dissatisfaction in all aspects of provided health care. The principal sources of discrimination identified by respondents were lack of money, social class and race. Gueveiva et al. [13] also found among the Brazilian WHS lack of money and social class as major factors of health care discrimination. It is important to note that the percentages of individuals who felt they had been treated worse than others on grounds of social exclusion were consistently higher among users of the public health care system, a practice that runs counter to the Bhato Pele (People first) guiding principles of the South African health care system. According to a qualitative study by Mashego and Peltzer [19] discrimination was also identified among primary public care users.

Unlike in some other studies [7], this study did not find significant associations between socio-demographic variables (age, sex and formal education) and patient satisfaction.

## Conclusion

Health care access, communication, autonomy, and discriminatory experiences were identified as priority areas for actions to improve responsiveness and patient satisfaction in South Africa. Implications for policymaking include that the result from the survey can be used to prioritize efforts when resources are limited. The data seem to provide a clear message to prioritize reforms that improve prompt attention, but not at the expense of patient dignity and communication, which may damage the acceptability of health services to users, and result in barriers to access.

### Study limitations

In this survey all respondents who had been hospitalized in the five years prior to the survey were asked about their most recent hospitalization, and all others were asked about their most recent ambulatory visit in the previous year. The result is that the set of respondents who answered the questions about ambulatory care is small and not representative of the general population, but rather of the population that had not been hospitalized during the previous five years. The cross-sectional study design did not permit an investigation of the cause-effect relationship between responsiveness and independent variables. Recall bias of study participants cannot be excluded, especially on the 5 year recall period for hospital admission.

## Competing interests

The author declares that they have no competing interests.

## Authors' contributions

KP designed the study, conducted the secondary analysis, and drafted and corrected the paper.

## Pre-publication history

The pre-publication history for this paper can be accessed here:


